# Comparative Proteomic Profiling of Secreted Extracellular Vesicles from Breast Fibroadenoma and Malignant Lesions: A Pilot Study

**DOI:** 10.3390/ijms23073989

**Published:** 2022-04-03

**Authors:** Katia Pane, Cristina Quintavalle, Silvia Nuzzo, Francesco Ingenito, Giuseppina Roscigno, Alessandra Affinito, Iolanda Scognamiglio, Birlipta Pattanayak, Enrico Gallo, Antonella Accardo, Guglielmo Thomas, Zoran Minic, Maxim V. Berezovski, Monica Franzese, Gerolama Condorelli

**Affiliations:** 1 IRCCS SYNLAB SDN, Via E. Gianturco 113, 80143 Naples, Italy; katia.pane@synlab.it (K.P.); silvia.nuzzo@synlab.it (S.N.); enrico.gallo@synlab.it (E.G.); 2 Institute for Experimental Endocrinology and Oncology (IEOS), National Research Council (CNR), Via Pansini 5, 80131 Naples, Italy; c.quintavalle@ieos.cnr.it; 3 Percuros BV, Eerbeeklaan 42, 2573 HT Den Haag, The Netherlands; francesco.ingenito@outlook.it (F.I.); giusy_roscigno@yahoo.it (G.R.); a.affinito@percuros.com (A.A.); 4 Department of Molecular Medicine and Medical Biotechnology, Federico II University of Naples, Via Pansini 15, 80131 Naples, Italy; iolanda.scognamiglio@gmail.com (I.S.); birlipta.pattanayak@gmail.com (B.P.); 5 Department of Pharmacy and Research Centre on Bioactive Peptides (CIRPeB), University of Naples “Federico II”, Via Mezzocannone 16, 80134 Naples, Italy; antonella.accardo@unina.it; 6 Breast Unit Clinica Mediterranea, Mediterranea Cardiocentro, Via Orazio 2, 80122 Naples, Italy; guglielmo.thomas@outlook.it; 7 John L. Holmes Mass Spectrometry Facility, Department of Chemistry and Biomolecular Sciences, University of Ottawa, Ottawa, ON K1N 6N5, Canada; zminic@uottawa.ca (Z.M.); maxim.berezovski@uottawa.ca (M.V.B.); 8 IRCCS Istituto Neurologico Mediterraneo (INM) Neuromed, Via Atinense 18, 86077 Pozzilli, Italy

**Keywords:** fibroadenoma, breast cancer, proteome, biomarker, EVs, extracellular vesicle, cell-to-cell signaling, disease monitoring, precision medicine, early diagnosis

## Abstract

Extracellular vesicles (EVs) shuttle proteins, RNA, DNA, and lipids crucial for cell-to-cell communication. Recent findings have highlighted that EVs, by virtue of their cargo, may also contribute to breast cancer (BC) growth and metastatic dissemination. Indeed, EVs are gaining great interest as non-invasive cancer biomarkers. However, little is known about the biological and physical properties of EVs from malignant BC lesions, and even less is understood about EVs from non-malignant lesions, such as breast fibroadenoma (FAD), which are clinically managed using conservative approaches. Thus, for this pilot study, we attempted to purify and explore the proteomic profiles of EVs from benign breast lesions, HER2+ BCs, triple–negative BCs (TNBCs), and continuous BC cell lines (i.e., BT-549, MCF–10A, and MDA-MB-231), combining experimental and semi-quantitative approaches. Of note, proteome-wide analyses showed 49 common proteins across EVs harvested from FAD, HER2+ BCs, TNBCs, and model BC lines. This is the first feasibility study evaluating the physicochemical composition and proteome of EVs from benign breast cells and primary and immortalized BC cells. Our preliminary results hold promise for possible implications in precision medicine for BC.

## 1. Introduction

Breast cancer (BC), a heterogeneous disease at the molecular level, is the most frequently diagnosed cancer in women. Patient survival rate is ~70–80% with early-stage, non-metastatic disease; in contrast, advanced stages of the disease are incurable [1]. Perou et al. classified BC into four subtypes (luminal A, luminal B, basal-like, and HER2-enriched) on the basis of the expression of 50 genes (PAM50) [2,3]. Traditionally, the diagnosis of BC is based on a triple-test clinical examination, imaging (usually mammography and/or ultrasonography), and needle biopsy [4]. However, tissue biopsies are very difficult to implement, and they are limited by tumor size. Therefore, there is great interest to further develop liquid biopsy methods and functional imaging for application in cancer diagnostics in a clinical setting.

Liquid biopsy is a new, low-cost, minimally invasive technique to diagnose different types of cancer. Circulating cells, microRNAs, circulating DNA, and extracellular vesicles (EVs) can be assessed in various biological fluids [5]. Genetic material isolated from blood, urine, and other biological liquids strongly reflect the clonal heterogeneity of the origin tissue [6], and circulating EVs have attracted much attention as promising biomarkers on account of their marked stability in non-physiological environments [7]. Indeed, tumor cells release a large amount of EVs, and profiling their proteome may reveal putative biomarker candidates for the early diagnosis of BC or for disease monitoring [8]. Mass-spectrometry-based proteomics is a cutting-edge technique for the identification and measurement of the relative abundance of proteins [9,10], and several studies have investigated the clinical potential of BC EV biomarkers from different sources [11,12]. However, precaution is necessary when translating results obtained from BC cell models to the clinical setting.

Many studies on BC have demonstrated that EVs have great potential as biomarkers because they can discriminate different BC subtypes, for example, triple–negative BC (TNBC) vs. ER/PR cell lines [11]. However, while methods for EV isolation and content assessment have been nicely described for those derived from biological fluids and immortalized cell lines from different subtypes of BC, there have been challenges in the isolation and physicochemical and cargo characterization of EVs from biopsy-derived primary cells, a fact that has hindered characterization. Moreover, benign lesions, such as breast fibroadenoma (FAD), may also secrete EVs, the functions of which are still largely unknown. For this reason, we set out to purify and characterize EV protein cargoes derived from benign and malignant breast biopsies and immortalized breast cell cultures (IBCCs). So far, no study has investigated the proteomic profile of EVs derived from IBCCs, comparing it with that from EVs from patient-specific cells, mainly because the purification and characterization of primary cell-derived EVs poses many challenges.

Large-scale production and high-throughput analysis of primary cell EVs from biopsy-derived tissues are affected by pre-processing factors for cell culturing and genomic testing. In fact, in the case of biopsy-derived primary cell cultures and EV purification, study is hindered by the need to enrich the tumor material, by tumor heterogeneity, and by the extraction method. Hence, knowledge on EV cargoes is lacking, especially on account of the small amount of starting material and the concurrent presence of normal and malignant tissue.

The current study is the first to investigate the feasibility of evaluating the physicochemical properties and the proteomes of EVs from benign lesions, malignant primary BC cells, and IBCCs. Prompted by the increasing findings on differences in EVs from different tissues of origin and their potential for BC diagnosis, monitoring, and response to therapy, we investigated the proteomic profile of breast FADs and the possible relationship with BC–derived EVs using high–throughput proteomics, comparing the EV proteomes of two patient-resected FADs and two distinct BC subtypes (i.e., HER2+ BC and TNBC) along with three IBCC lines (i.e., BT–549, MCF–10A, and MDA–MB–231).

## 2. Materials and Methods

### 2.1. Cell Lines and Primary Cultures

Primary epithelial BC and FAD cultures were prepared from biopsies obtained from patients at Clinica Mediterranea S.p.A, as previously reported [13]. Our study included two biological replicates and three technical replicates for each patient-derived EV preparation, whereas there were three technical replicates for each model-derived EV preparation.

Cell cultures were grown in Dulbecco’s modified Eagle’s medium (DMEM)/Nutrient F12-Ham (DMEM-F12) supplemented with 10% fetal bovine serum (FBS) (Sigma-Aldrich, Milan, Italy). Continuous cell lines were purchased from ATCC (LG Standards, Milan, Italy) and grown in the following media: Roswell Park Memorial Institute medium (RPMI) for human breast BT549 and MDA-MB-231 cells; DMEM/F12 supplemented with EGF (20 ng/mL), hydrocortisone (0.5 μg/mL), cholera toxin (100 ng/mL), insulin (10 μg/mL), and horse serum (5%) for human breast MCF–10A cells. All cells were grown at 37 °C in 95% air and 5% CO_2_.

### 2.2. Purification of Extracellular Vesicles

EVs were isolated from the culture media of cells grown in serum–free medium supplemented with 10% Exo-FBS (FBS depleted of EVs, SBI, System Biosciences, CA, USA) in 150 mm plates (15 mL medium volume) with Cell Culture Media Extracellular Vesicle Purification Kits (Norgen, Biotek Corp, Ontario, Canada), according to the manufacturer’s instructions.

### 2.3. Dynamic Light Scattering (DLS) Measurements

Mean diameter of EVs from MDA-MB-231 cells, HER2+ BC patient #37, FAD patient #44, and TNBC patient #148 was measured using DLS on a Zetasizer Nano ZS 326 (Malvern Instruments, Westborough, MA, USA). Instrumental settings for the measurements were a backscatter detector at 173° in automatic modality, room temperature, and a disposable sizing cuvette as cell. DLS measurements in triplicate were carried out on aqueous samples after centrifugation at room temperature at 13,000 rpm for 5 min.

### 2.4. Scanning Electron Microscopy (SEM)

Morphological analysis of EVs from MDA-MB-231 cells and patients #37, #44, and #148 was carried out using field emission SEM (Phenom XL, Alfatest, Milan, Italy). To remove salt, the EV solution was twice dialyzed against water (cut-off membrane = 3500 Da). A 10 μL sample of the resulting solution was drop-casted on an aluminum stub and air-dried. A thin coat of gold and palladium was sputtered at a current of 25 mA for 75 s. The sputter-coated samples were then introduced into the specimen chamber and the images were acquired at an accelerating voltage of 10 kV, spot 3, through the Secondary Electron Detector (SED) [14].

### 2.5. Liquid Chromatography Tandem Mass Spectrometry (LC-MS/MS)

In-solution digestion of protein was performed, as previously described, in the presence of 0.1% n-dodecyl β-D-maltoside (DDM) [15]. Then, samples were analyzed using an Orbitrap Fusion (Thermo Fisher Scientific, Ontario, Canada) coupled to an Ultimate 3000 nanoRLSC (Dionex, Thermo Fisher Scientific). Peptides were separated on an in-house packed column (Polymicro Technology, CM Scientific Ryefield Ltd, Republic of Ireland), 15 cm × 70 μm ID, Luna C18(2), 3 μm, 100 Å (Phenomenex, CA, USA), employing a water/acetonitrile/0.1% formic acid gradient. Samples were loaded onto the column for 105 min at a flow rate of 0.30 μL/min. Peptides were separated using 2% acetonitrile in the first 7 min and then using a linear gradient from 2 to 38% of acetonitrile for 70 min, followed by a gradient from 38 to 98% of acetonitrile for 9 min, then at 98% of acetonitrile for 10 min, followed by a gradient from 98 to 2% of acetonitrile for 3 min and a wash at 2% of acetonitrile for 10 min. Eluted peptides were directly sprayed into the mass spectrometer using positive electrospray ionization (ESI) at an ion source temperature of 250 °C and an ion spray voltage of 2.1 kV. The Orbitrap Fusion Tribrid was run in top speed mode. Full-scan MS spectra (*m*/*z* 350–2000) were acquired at a resolution of 60,000. Precursor ions were filtered according to monoisotopic precursor selection, charge state (+2 to + 7), and dynamic exclusion (30 s with a ±10 ppm window). The automatic gain control settings were 4 × 10^5^ for full FTMS scans and 1x10^4^ for MS/MS scans. Fragmentation was performed with collision-induced dissociation (CID) in the linear ion trap. Precursors were isolated using a 2 *m*/*z* isolation window and fragmented with a normalized collision energy of 35%.

### 2.6. Database Searches and Bioinformatics Analyses

Firstly, Proteome discoverer 2.1 (Thermo Fisher Scientific) was used for protein identification. The precursor mass tolerance was set at 10 ppm and 0.6 Da mass tolerance for fragment ions. Search engine: SEQUEST-HT implemented in Proteome Discovery was used for all MS raw files. Search parameters were set to allow for dynamic modification of methionine oxidation, acetyl on N-terminus, and static modification of cysteine carbamidomethylation. The search database consisted of nonredundant/reviewed human (20,326 proteins) protein sequences in FASTA file format from the UniProt/SwissProt database. The FDR was set to 0.05 for both peptide and protein identification.

Secondly, MaxQuant computational platform [16] was used to perform proteomic data analyses as previously described [15]. Default parameters were used if not otherwise described. Trypsin and LysC, C-terminal cleavage at lysine and arginine, were set as digestion enzymes to generate peptides of at least 7 amino acids with a maximum of 2 missed cleavages. Identified peptides had an initial precursor mass deviation of up to 10 ppm and a fragment mass deviation of 0.6 Da. The false discovery rate (FDR) for peptides and proteins of 0.05 was determined using a reverse sequence database. Label-free protein quantification (LFQ) values were obtained through the MaxLFQ algorithm, considering only unique peptides. A contaminants database provided by MaxQuant was used. Downstream analysis included the combination of results from ProteomeDiscover and MaxQuant. We identified for each condition a high confident protein set resulting from the overlapping proteins identified in all three technical replicates. We used Exocarta Database for the assessment of any known exosomal proteins within primary and model cell line EV cargoes. Protein subcellular locations were retrieved using Gene Ontology (GO) and Ingenuity Pathways analysis (IPA) QIAGEN software [17]. In IPA, we carried out pathway enrichment analyses by filtering the enrichment score at a threshold of ≥5 (Fisher’s exact right-tailed test). A heatmap generated from R software (version 3.6.3) [18] displayed the log-transformed average intensities of non-zero protein mean values across three replicates of breast primary and model cell biosamples. Dendrograms on the left side and top showed the abundance patterns clustered by row and column means, respectively.

### 2.7. Western Blot Analysis

EVs were lysed in an RIPA buffer (Thermo Scientific, Milan, Italy Pierce RIPA buffer) containing protease inhibitor (Roche, Milan, Italy Protease inhibitor cocktail tablets) and phosphatases inhibitor (Sigma Aldrich Milan, Italy, Phosphatase inhibitor cocktail 3) for 2 h in ice and the protein concentration was determined using the Bradford assay (Bio-Rad, Milan, Italy Protein Assay Dye Reagent Concentrate). Then, 16 μg of protein was separated using 10% SDS-PAGE and blotted onto a nitrocellulose membrane (GE Healthcare, Milan Italy Amersham Protran 0.45 µm NC ). The membranes were stained with a Ponceau S solution (Sigma, P7170-1L) and then washed with Tris-buffered saline containing 0.1% Tween-20 (T-TBS). After blocking with 5% no-fat dry milk in T-TBS, the membranes were incubated at 4 °C overnight with the following primary antibodies: anti-COL1A2 (Abcam, Cambridge, UK ab96723 1:1000), anti-Cytokeratin 2e (KRT2 Abcam, ab170106 1:800), anti-Alix (Santacruz Biotechnology, Texas, USA, 1A12 1:250), anti-Calnexin (Abcam ab10286 1:1000), and anti-TSG101 (Abcam ab83 1:500). Detection was performed using peroxidase-conjugated secondary antibodies using the ultra-enhanced chemiluminescence system (Thermo Scientific, Super Signal West Femto Maximum Sensitivity Substrate #34095).

## 3. Results

### 3.1. Study Design and Sample Description

**Figure 1:**
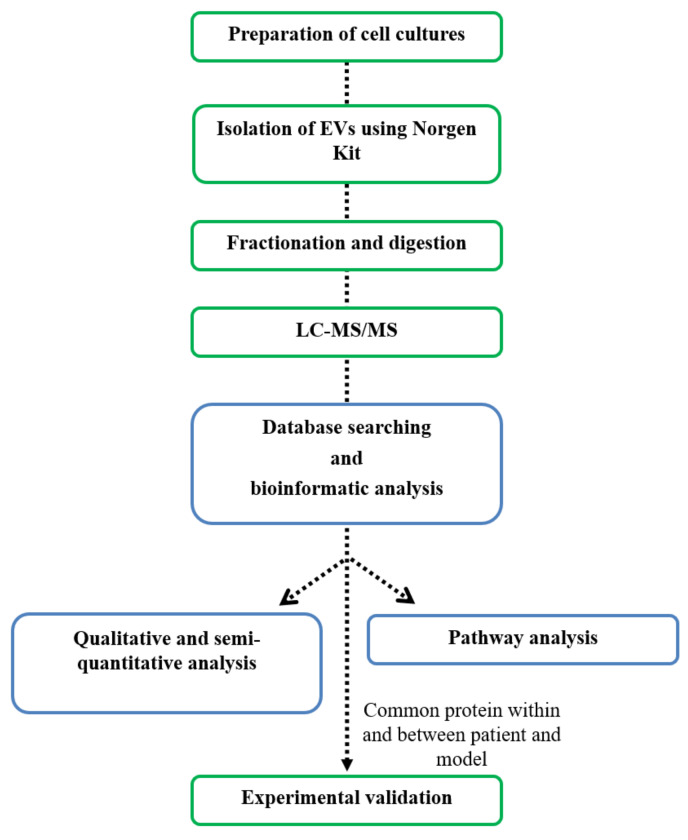
Workflow followed for this study. Schematic representation of the experimental phase (green box) and the computational phase (blue box). Arrows represent outputs.

We performed MS-based proteomic analyses on all samples, applying the methodological workflow shown in Figure 1.

### 3.2. Characterization of EVs from Primary and IBCC Lines

EVs were isolated from primary cells harvested from biopsies and from the IBCC lines BT-549, MCF–10A, and MDA-MB-231; biopsies were from two breast FADs, two HER2+ BCs, and two TNBCs (Table 1).

**Table 1:** Clinical characteristics of patients and samples used in this study.

Patient	Sample #37	Sample #46	Sample #72	Sample #44	Sample #170	Sample #148
Median age at diagnosis (years)	49.5		43		53.5	
Age at diagnosis (years)	66	33	44	42	56	51
Histological type	Ductal infiltrating carcinoma (NOS)	Ductal infiltrating carcinoma high grade	Fibroadenoma	Fibroadenoma with adenosi	Ductal infiltrating carcinoma (NOS) CK19 (+++)	Lobular infiltrating carcinoma, poorly differentiated with 10% of lobular neoplasia in situ with high grade, E-cadherin negative
Tumor stage	pT1c	pT2	_	_	pT2	pT2
Grade	pG3	pG3	_	_	pG3	pG3
Lymph node	pN1a	pN1a	_	_	0	0
ER/PR/HER2 status (positivity)	ER − (0)/PR + (<5)/HER2 + (2+)	ER +(10)/PR + ^a^/HER2 + (3+)	_	_	ER − (0)/PR − (0)/HER2 − (0)	ER − (0)/PR − (<5)/HER2 − (0)
ki 67 status (positivity)	High (30)	High (50)	_	_	High (80)	High (45)
Subtype	HER2+ BC	HER2+ BC	FAD	FAD	TNBC	TNBC
Corresponding cell model	BT-549		MCF10-A		MDA-MB-231	

^a^ Focal positivity. We assessed the morphology of EVs from MDA-MB-231 cells and from primary cell cultures from patients #37, #44, and #148 (at 10 and 3 µm) using SEM (Figure 2a–d and Figure S1, respectively); size distribution in aequeous solution was assessed using DLS (Figure 2e–h).

Sizes ranged from about 300 nm to 350 nm. All diffusion coefficients, mean diameters (calculated using the Stokes–Einstein equation), and the polydispersity indexes are reported in Table 2. SEM measurements were in good agreement with the structural information obtained using DLS characterization.

### 3.3. Qualitative and Semi-Quantitative MS-Based Proteomic Profiling

To acquire insight into the proteomic profile of BC EVs, all samples were analyzed using high-resolution LC-MS/MS (Table 1). To identify a protein set with high confidence, we used the combined outputs of two search engines: Proteome Discoverer and MaxQuant. FDR was set at <0.05 for filtering cutoff (third analysis combination). Considering all three runs, there were 127 proteins in HER2+ EVs, 132 proteins in FAD EVs, and 146 proteins in TNBC EVs. Similarly, BT-549, MCF–10A, and MDA-MB-231 cell EVs had 161, 278, and 172 proteins, respectively.

### 3.4. Pathway Analysis

We investigated the potential involvement of EV proteins in biological pathways (Figure S2) by leveraging IPA bioinformatics software [17]. We selected the most statistically significant biological processes associated with IBCC and primary cell EVs. The protein cargoes of primary cell EVs were enriched in signaling pathways involved in phagosome formation, remodeling of epithelial adherents’ junctions, and clathrin-mediated endocytosis signaling, among others. Thus, the profiles reflected multiple cell-to-cell communication processes and endocytosis signaling as likely expected for the EVs (Figure S2A). Of note, the EV proteome of FADs regulated many pathways also identified in BC EVs (Figure S2A).

**Figure 3:**
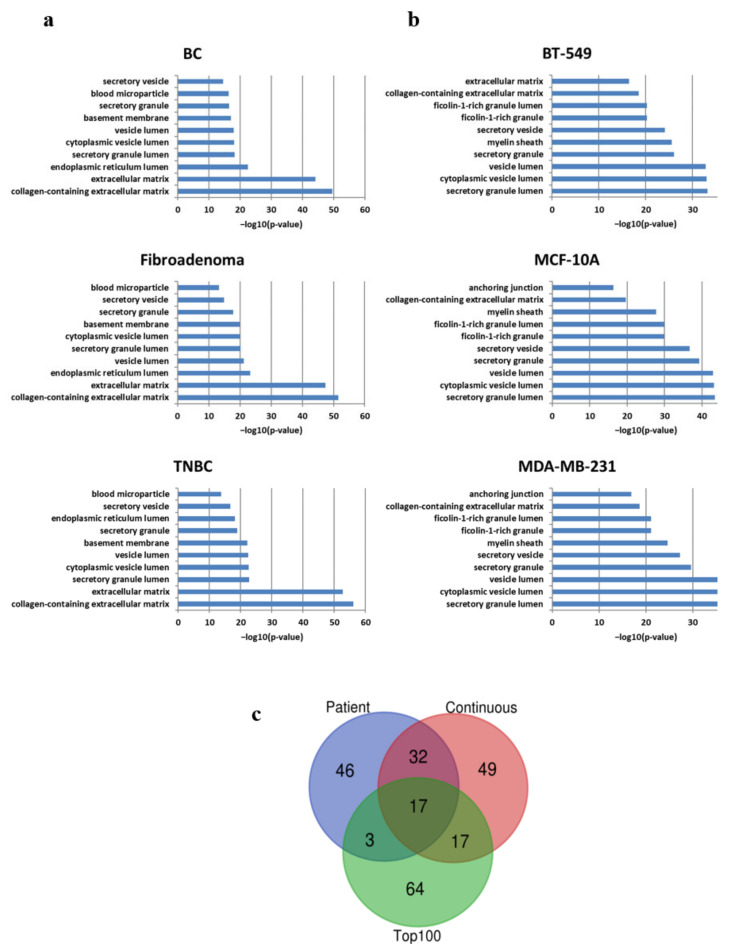
Proteome-wide identification of patient and model cell line EV cargoes. (**a**) Gene ontology cellular component of patient-derived EVs. (**b**) Gene ontology cellular component of continuous-derived EVs. (**c**) Venn diagrams of overlapping protein for patient and model cells including exosomal markers (ExocartaDB TOP 100 table).

We also performed IPA to discriminate enriched pathways in the proteomes of IBCC -derived EVs (Figure S2B). Compared with EVs derived from BT-549 and MDA-MB-231 cells, MCF–10A cell EVs were enriched in fewer statistically significant pathways, such as phagosome maturation, remodeling of epithelial adherents’ junctions, clathrin-mediated endocytosis signaling, and protein ubiquitination (Figure S2B). Indeed, IBCC and primary cell EV cargoes included members of vanin, tubulin, collagen, and actin cytoskeleton protein families, according to gene ontology (GO) cellular compartment annotations (Figure 3a,b, respectively).

Taken together, pathway analysis and subcellular location using GO revealed that the majority of EV proteins were involved in multiple dynamic cellular processes, including E-cadherin signaling on neighboring cells, tight and adherents junction signaling, and EV formation and release into the extracellular space. These findings supported the secretory nature of EVs isolated from both sample types, namely IBCCs and primary cells.

To gain additional biological insight, we compared patient–derived (*n* = 98) and IBCC–derived (*n* = 115) EV proteins, finding 49 molecules that were shared between the two (Figure 3c and Table S1): among these, 17 proteins were well-known EV molecules, as reported in Exocarta Top 100 most recurrent EV markers (Figure 3c and Table S1). Some play key roles in glycolysis (ALDOA, GAPDH, ENO1, PGK1, PKM, TPI1), others are major microtubule constituents (TUBA1A, TUBA1B, TUBA1C), and some are involved in remodeling of epithelial adherents’ junctions or other dynamic processes, such as the building of structural cytoskeleton (ACTAB, ACTG1, ACTA1).

### 3.5. Protein Abundance Patterns and In Vitro Investigation

**Figure 4:**
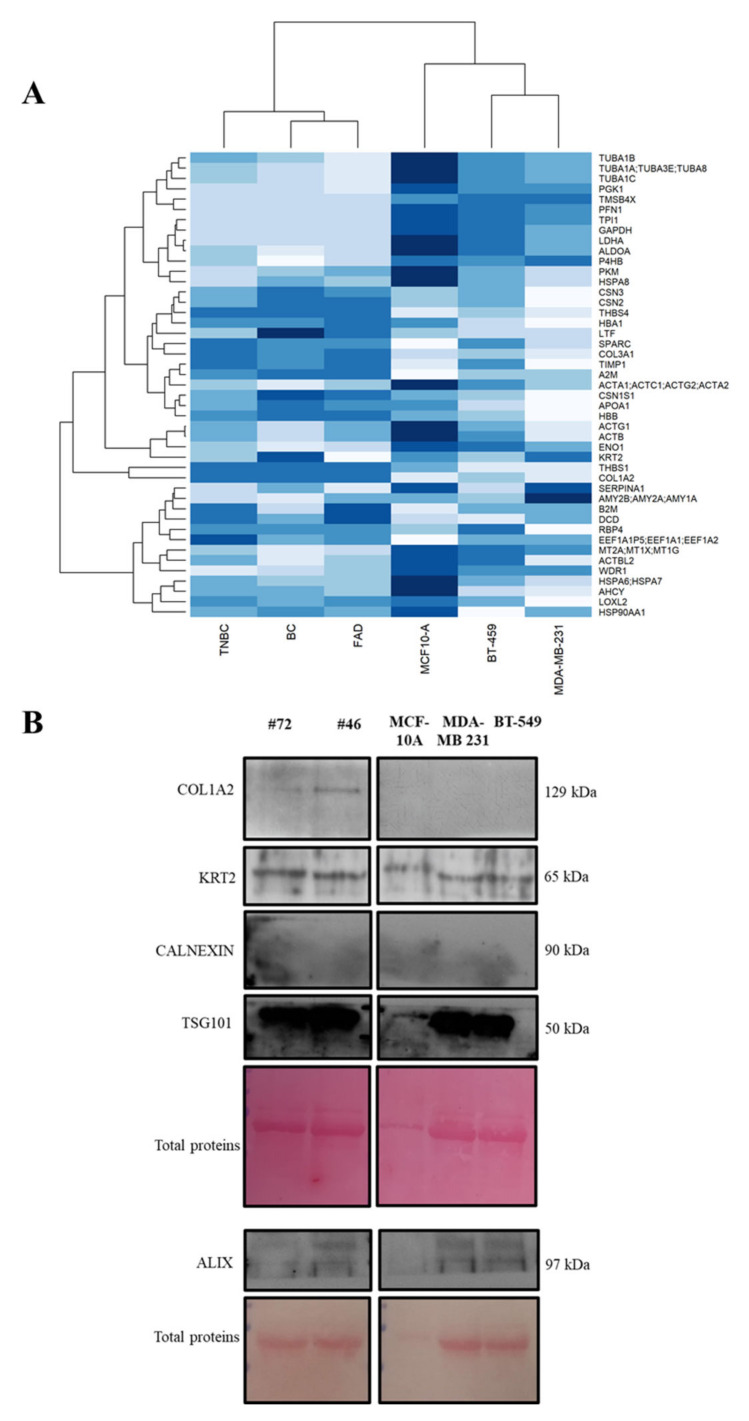
Common protein abundance patterns in EVs from primary BC cells and models. (**A**) Heatmap of non-zero protein mean abundance (log-transformed average values, in the rows) across breast tissue/cell samples (columns, from left to right side), respectively. Color intensity from low abundance (white) to high abundance (blue). BC, breast cancer; TNBC, triple–negative; FDA, fibroadenoma. (**B**) Western blot analysis of COL1A2, KRT2, CALNEXIN, TSG101, and ALIX in EVs from FAD patient #72, HER2+ BC patient #46, and the three IBCC lines. The heatmap shows the abundance patterns for non-zero mean abundance proteins (rows) across the different samples (columns). We found different patterns of abundance for most EV proteins in primary cells vs. IBCCs. Proteins with the most similar pattern across the two were collagen type I alpha 2 chain (COL1A2) and keratin type II (KRT2), an intermediate filament protein member. The ranked relative abundance (log LFQ intensity) of the identified protein groups is shown in Figure S3.

Ultrahigh-resolution LC-MS/MS and proteomic characterization of EVs from primary cells and IBCCs led us to identify a common signature of 49 proteins across the distinct breast tissue/cell types. We profiled the average abundance of the 49 common proteins across all the cell types investigated in our study (Figure 4A), carrying out clustering analysis to assess the abundance patterns.

To confirm that our semiquantitative proteomic approach was able to discriminate differences between IBCCs and primary cell EVS, we focused on COL1A2 and KRT2 expression and evaluated their levels in patient-derived lines #72 and #46, comparing them with those in IBCCs.

COL1A2, was mainly expressed in patient-derived EVs, with no expression detected in EVs from IBCCs, whereas KRT2 was equally expressed in both cell groups (Figure 4B).

Of note, the expression of Alix and TSG101, two positive exosomal marker, and the absence of Calnexin indicated an enrichment of exosomal fraction in our EVs preparation.

## 4. Discussion

Several recent studies have highlighted the importance of identifying subtype-specific BC biomarkers in EVs from cultured cells and patient-derived cells [11]. Indeed, the identification of biomarkers could lead to the development of powerful, non–invasive tools for the detection and classification of BCs. Proteomic analysis is a widely used method for the identification of EV protein candidates from a variety of solid tumors, including from breast, colon, blood, and lung [19,20,21]. To the best of our knowledge, the current study is the first aimed at evaluating the physicochemical composition and proteomic profile of EVs from benign breast tumor, primary cells from malignant BC, and immortalized breast cell lines: a high-throughput proteomic approach was used to explore the EV proteome of two FADs, two distinct BC subtypes (i.e., HER2+ and TNBC), and the IBCC lines BT-549, MCF–10A, and MDA-MB-231. Pathway analyses and subcellular location revealed that most EV proteins identified from the primary and immortalized lines are involved in multiple dynamic cellular processes, such as cellular communication and EV formation and release into the extracellular space. We also obtained proof of concept that it is possible to generate a common EV protein profile for primary and immortalized cells. Indeed, the computational robustness of our approach—which employed two search engines and stringent filtering criteria—allowed us to identify a protein set with high confidence. We found a signature of 49 common proteins in EVs from benign and malignant lesions: the proteins were involved in glycolysis, major microtubule formation, and remodeling of epithelial adherents’ junctions. We also analyzed the mean abundance of most proteins in patient EVs, comparing data with those from breast cell lines: we found a relative abundance of KRT2. However, it was quite difficult to assess external contamination of our samples with this protein [22]; indeed, some KRT family members are commonly and universally found in body fluids, such as serum and saliva [23,24].

Hoshino et al. [25] highlighted differences in EV proteins based on the tissue of origin; more importantly, they found that markers of cell line EVs could not be immediately translated to patient-derived EVs. Nevertheless, our findings clearly indicate that there could be a common EV-marker signature for IBCC and patient–derived cell lines.

It is still debated whether FAD is a risk factor for BC development [26,27,28]: some authors have reported increased relative risk of subsequent BC in patients with FAD [29,30], but a very recent Mayo Clinic study has highlighted that even if complex FAD is present, it does not confer increased risk of BC [31]. On this point, our finding of a high similarity in the proteomic profiles of EVs from FAD and BC cell lines clearly indicates that precaution is necessary when classifying benign lesions as being non-tumoral.

In this study, we used LC-MS/MS proteomics and database searches for peptide identification, approaches used widely over the last decades for multiple applications [19,20,21]. High-throughput proteomics is revolutionizing laboratory medicine, although some capabilities still need to evolve [32]. For instance, sample preparation is one of the most challenging procedures for reliable and accurate analyte determination and precise peptide identification through tandem MS spectra [10,33,34].

Further, in proteomics analysis, membrane proteins are traditionally under-represented, mainly due to their low solubility [35]. In a previous investigation, different detergents (DDM, Triton X-100, and Digitonin) were used to analyze EVs proteome [15]. DDM was found to be the best detergent to recover more EVs proteins. Here, we used DDM as an LC-MS-compatible detergent for the digestion of samples. However, proteins vary in size, charge, and hydrophobicity, and there is no specific detergent for the solubilization of all proteins [36]. Therefore, we expect that some relatively hydrophobic proteins were not well solubilized and, consequently, were not identified. For this reason, it will be necessary to develop new methods for the solubilization of EVs in the future.

The present study paves the way for increasing analysis complexity, querying, and the combination of results from multiple search engines, a most powerful approach [10,33,34], to generate a highly confident proteome set for individual samples. EV biogenesis, isolation, and proteomics from patient-derived and continuous cell lines is still a poorly explored world that could lead to a plethora of therapeutic and diagnostic applications [5,37,38]. Our analysis pipeline has shed light on a common signature of 49 proteins in EVs from primary and model BC cells: the two proteins that were studied for experimental validation (COL1A2 and KRT2) agreed with the in-silico findings. However, this preliminary study does suffer from poor data generalization because we could characterize EV cargoes from only six patient-specific mammary epithelial cells on account of the challenges associated with collection of clinical samples and the isolation of patient–derived EVs. Nevertheless, we describe for the first time a remarkable overlap between EV proteins from BC models and patient-derived BC cell lines. Further investigation aimed at the identification of clinically relevant BC EV biomarkers is warranted.

## 5. Conclusions

EVs are produced by all cell types and mediate intercellular communication by carrying proteins, DNA, RNA, lipids, and microRNAs involved in physiological and pathophysiological processes. Recent studies have reported on EV biomarkers (microRNAs and proteins) associated with a more aggressive phenotype in multiple cancers. For instance, some BC EV proteins allow BC cells to communicate with the surrounding “normal” cells, promoting invasion and migration of BC cells and/or pre-metastatic niche establishment. Thereby, comprehensive molecular analysis of EV cargoes would deepen insight into their mechanisms of action, possibly with huge clinical implications in cancer diagnosis and prognosis. Currently, innovative high-throughput techniques can unveil EV origins and critical roles. In our study, we carried out mass spectrometry-based proteomics analysis, which is revolutionizing laboratory medicine, although some capabilities still need to evolve in this field. At present, major challenges are due to protocol differences in the isolation of EV populations, the analytical platforms used, and the difficulties encountered when performing studies on large clinical cohorts. The latter issue has also affected the generalization of our findings. Indeed, we will need to extend our investigation before ascertaining definitive specific signatures for BC EVs. To this end, studies performed on large clinical cohorts, with harmonization of protocols across laboratories, will be helpful in bringing advances to the EV field and discovering non-invasive and reliable biomarkers for better BC diagnosis and monitoring.

## Figures and Tables

**Figure 2:**
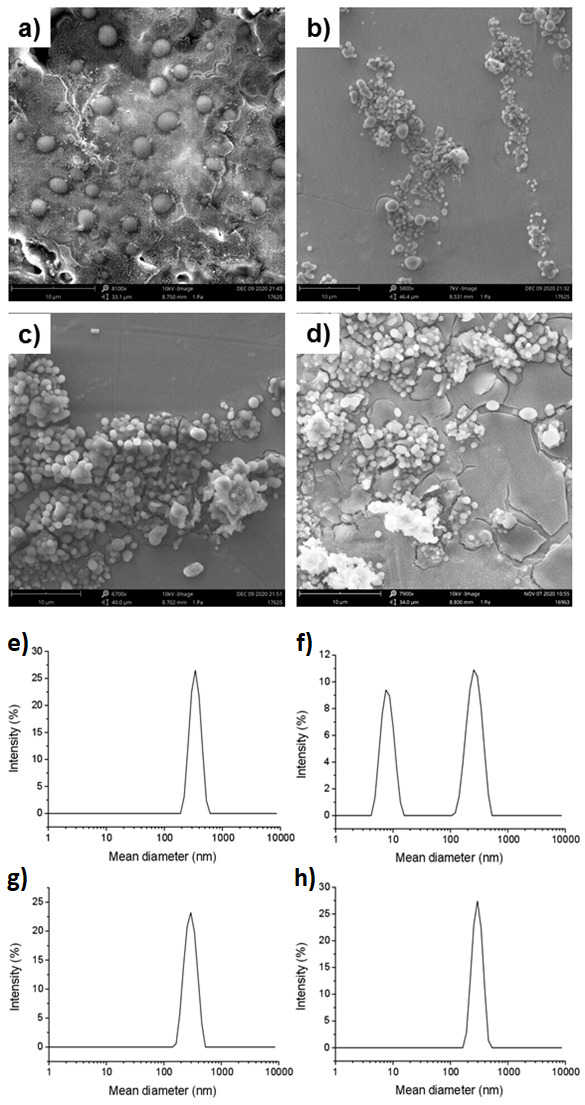
Characterization of primary and model cell line EVs. SEM micrographs of EVs drop-casted on aluminum stubs: (**a**) EVs from patient HER2+ BC #37; (**b**) EVs from FAD patient #44; (**c**) EVs from TNBC patient #148; and (**d**) MDA-MB-231 cell EVs. Magnification and scale bar for all samples is 7900×, 10 µm. Dynamic light scattering intensity profile of EVs in aqueous solution: (**e**) #37 EVs; (**f**) #44 EVs; (**g**) #148 EV; and (**h**) MDA-MB-231 EVs.

**Table 2:** Diffusion coefficients (D), mean diameters, and polydispersity indexes (PDI) from DLS measurements for the EVs studied.

Sample	Mean Diameter (nm) ± S.D.	PDI	(D ± S.D.) * 10^−12^ m^2^ s^−1^
#37	346 ± 70	0.356	1.15 ± 0.23
#44	270 ± 74 8 ± 2	0.395	1.47 ± 0.40 49.6 ± 12.4
#148	298 ± 64	0.365	1.33 ± 0.29
MDA-MB-231	295 ± 58	0.310	1.39 ± 0.27

## Data Availability

The original contributions presented in the study are included within the manuscript and in Supplementary Materials; any additional data can be obtained upon request to the corresponding authors.

## Supplementary Materials

The following supporting information can be downloaded at: https://www.mdpi.com/article/10.3390/ijms23073989/s1.

## Abbreviations

BC	breast cancer
DDM	n-dodecyl β-D-maltoside
DLS	dynamic light scattering
ESI	electrospray ionization
ER	estrogen receptor
EVs	extracellular vesicles
FAD	fibroadenoma
FDR	false discovery rate
HER2	human epidermal growth factor receptor 2
IBCC	immortalized breast cell culture
PR	progesterone receptor
SED	secondary electron detector
SEM	scanning electron microscopy
TNBC	triple–negative breast cancer
