# Supplementary material for: Comparative Proteomic Profiling of Secreted Extracellular Vesicles from Breast Fibroadenoma and Malignant Lesions: A Pilot Study
Source: Int J Mol Sci. 2022 Apr 3;23(7):3989. doi: 10.3390/ijms23073989 (PMC8999736; doi:10.3390/ijms23073989)
Supplement: Supplementary file 1 [file ijms-23-03989-s001.zip › ijms-1605626-supplementary.pdf]

International Journal of
Molecular Sciences

MDPI

Supplementary Material

1.1 Supplementary Tables

Table S1 attached to Figure 3

1.2 Supplementary Figures

Figure S1 attached to Figure 2

Figure S2 attached to Figure 3

Figure S3 attached to Figure 3

International Journal of *Molecular Sciences*

MDPI

Table S1. 49 common protein annotations between patient–derived and immortalized breast cell cultures (IBCC) (continuous cells).

Gene stable ID	Protein name	Protein_ IDs	Location	Type(s)	Gene description	Gene_ID
ENSG00000175899	A2M	P01023	Extracellular Space	transporter	alpha-2-macroglobulin	7
ENSG00000143632	ACTA1;ACTC1;ACTG2;ACTA2	P68133;P68032;P63267;P62736	Cytoplasm	other	actin alpha 1. skeletal muscle	129
ENSG00000075624	ACTB	P60709;Q6S8J3;A5A3E0;P0CG38;Q9BYX7;P0CG39	Cytoplasm	other	actin beta	132
ENSG00000169067	ACTBL2	Q562R1	Nucleus	other	actin beta like 2	17780
ENSG00000184009	ACTG1	P63261	Cytoplasm	other	actin gamma 1	144
ENSG00000101444	AHCY	P23526	Cytoplasm	enzymes	adenosylhomocysteinase	343
ENSG00000149925	ALDOA	P04075	Cytoplasm	enzymes	aldolase. fructose-bisphosphate A	414
ENSG00000240038	AMY2B;AMY2A;AMY1A	P19961;P04746;P04745	Extracellular Space	enzymes	amylase alpha 2B	478
ENSG00000118137	APOA1	P02647	Extracellular Space	transporter	apolipoprotein A1	600
ENSG00000273686	B2M	P61769	Plasma Membrane	transmembrane receptor	beta-2-microglobulin	914
ENSG00000214324	C3orf56	Q8N813	Other	other	chromosome 3 open reading frame 56	32481
ENSG00000164692	COL1A2	P08123	Extracellular Space	other	collagen type I alpha 2 chain	2198
ENSG00000168542	COL3A1	P02461	Extracellular Space	other	collagen type III alpha 1 chain	2201

Gene stable ID	Protein name	Protein_IDs	Location	Type(s)	Gene description	Gene_ID
ENSG00000138495	COX17	Q14061	Cytoplasm	enzymes	cytochrome c oxidase copper chaperone COX17	2264
ENSG00000126545	CSN1S1	P47710	Extracellular Space	other	casein alpha s1	2445
ENSG00000135222	CSN2	P05814	Extracellular Space	kinase	casein beta	2447
ENSG00000171209	CSN3	P07498	Extracellular Space	other	casein kappa	2446
ENSG00000161634	DCD	P81605	Extracellular Space	other	dermcidin	14669
ENSG00000196205	EEF1A1P5;EEF1A1;EEF1A2	Q5VTE0;P68104;Q05639	Extracellular Space	other	eukaryotic translation elongation factor 1 alpha 1 pseudogene 5	3200
ENSG00000074800	ENO1	P06733;P13929	Cytoplasm	enzymes	enolase 1	3350
ENSG00000177294	FBXO39	Q8N4B4	Other	other	F-box protein 39	28565
ENSG00000111640	GAPDH	P04406;O14556	Cytoplasm	enzymes	glyceraldehyde-3-phosphate dehydrogenase	4141
ENSG00000206172	HBA1	P69905	Extracellular Space	transporter	hemoglobin subunit alpha 1	4823
ENSG00000244734	HBB	P68871;P02042;P69892;P69891;P02100	Cytoplasm	transporter	hemoglobin subunit beta	4827
ENSG00000080824	HSP90AA1	P07900;Q14568;Q58FF8;Q58FF7;Q58FG1	Cytoplasm	enzymes	heat shock protein 90 alpha family class A member 1	5253
ENSG00000173110	HSPA6;HSPA7	P17066;P48741	Nucleus	enzymes	heat shock protein family A (Hsp70) member 6	5239

Gene stable ID	Protein name	Protein_ IDs	Location	Type(s)	Gene description	Gene_ID
ENSG00000109971	HSPA8	P11142; P54652	Cytoplasm	enzymes	heat shock protein family A (Hsp70) member 8	5241
ENSG00000138182	KIF20B	Q96Q89	Nucleus	enzymes	kinesin family member 20B	7212
ENSG00000172867	KRT2	P35908	Cytoplasm	other	keratin 2	6439
ENSG00000134333	LDHA	P00338; Q6ZMR3;P07864	Cytoplasm	enzymes	lactate dehydrogenase A	6535
ENSG00000134013	LOXL2	Q9Y4K0	Extracellular Space	enzymes	lysyl oxidase like 2	6666
ENSG00000012223	LTF	P02788	Extracellular Space	peptidase	lactotransferrin	6720
ENSG00000125148	MT2A;MT1X;MT1G	P02795; P80297; P13640; P80294; P04731	Cytoplasm	other	metallothionein 2A	7406
ENSG00000185624	P4HB	P07237	Cytoplasm	enzymes	prolyl 4-hydroxylase subunit beta	8548
ENSG00000108518	PFN1	P07737	Cytoplasm	other	profilin 1	8881
ENSG00000102144	PGK1	P00558	Cytoplasm	kinase	phosphoglycerate kinase 1	8896
ENSG00000067225	PKM	P14618	Cytoplasm	kinase	pyruvate kinase M1/2	9021
ENSG00000138207	RBP4	P02753	Extracellular Space	other	retinol binding protein 4	9922
ENSG00000197249	SERPINA1	P01009	Extracellular Space	other	serpin family A member 1	8941
ENSG00000113140	SPARC	P09486	Extracellular Space	other	secreted protein acidic and cysteine rich	11219
ENSG00000137801	THBS1	P07996	Extracellular Space	other	thrombospondin 1	11785
ENSG00000113296	THBS4	P35443	Extracellular Space	other	thrombospondin 4	11788

Gene stable ID	Protein name	Protein_IDs	Location	Type(s)	Gene description	Gene_ID
ENSG00000102265	TIMP1	P01033	Extracellular Space	cytokine	TIMP metallopeptidase inhibitor 1	11820
ENSG00000205542	TMSB4X	P62328	Cytoplasm	other	thymosin beta 4 X-linked	11881
ENSG00000111669	TPI1	P60174	Cytoplasm	enzymes	triosephosphate isomerase 1	12009
ENSG00000167552	TUBA1A;TUBA3E;TUBA8	Q71U36;P0DPH8;P0DPH7;Q6PEY2;Q9NY65	Cytoplasm	other	tubulin alpha 1a	20766
ENSG00000123416	TUBA1B	P68363;A6NHL2	Cytoplasm	other	tubulin alpha 1b	18809
ENSG00000167553	TUBA1C	Q9BQE3	Cytoplasm	other	tubulin alpha 1c	20768
ENSG00000071127	WDR1	O75083	Extracellular Space	other	WD repeat domain 1	12754

International Journal of *Molecular Sciences*

MDPI

Figure S1 attached to Figure 2

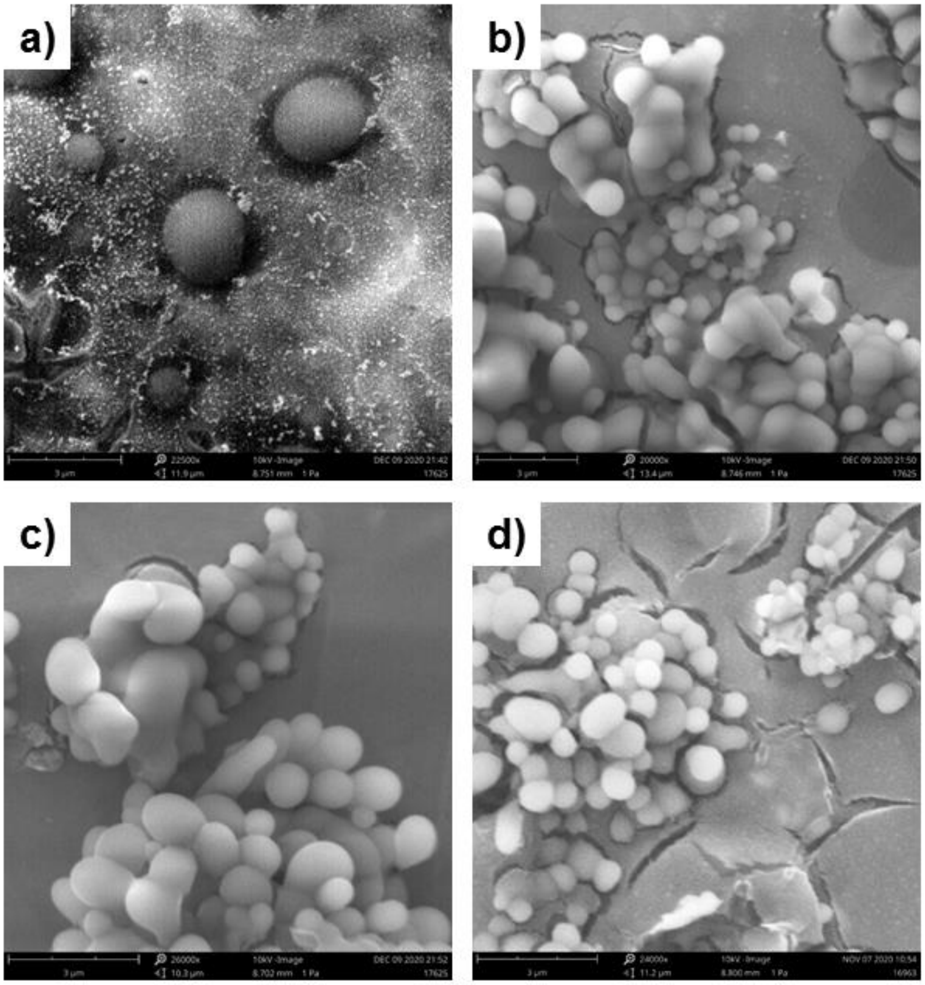

Figure S1. SEM micrographs for exosomes drop-casted on aluminium stubs: a) exosomes 37; b) exosomes 44, c) exosomes 148 and d) exosomes MDA-M3231. Magnification and scale bar for all samples is 24000x, 3μm.

Figure S2 attached to Figure 3

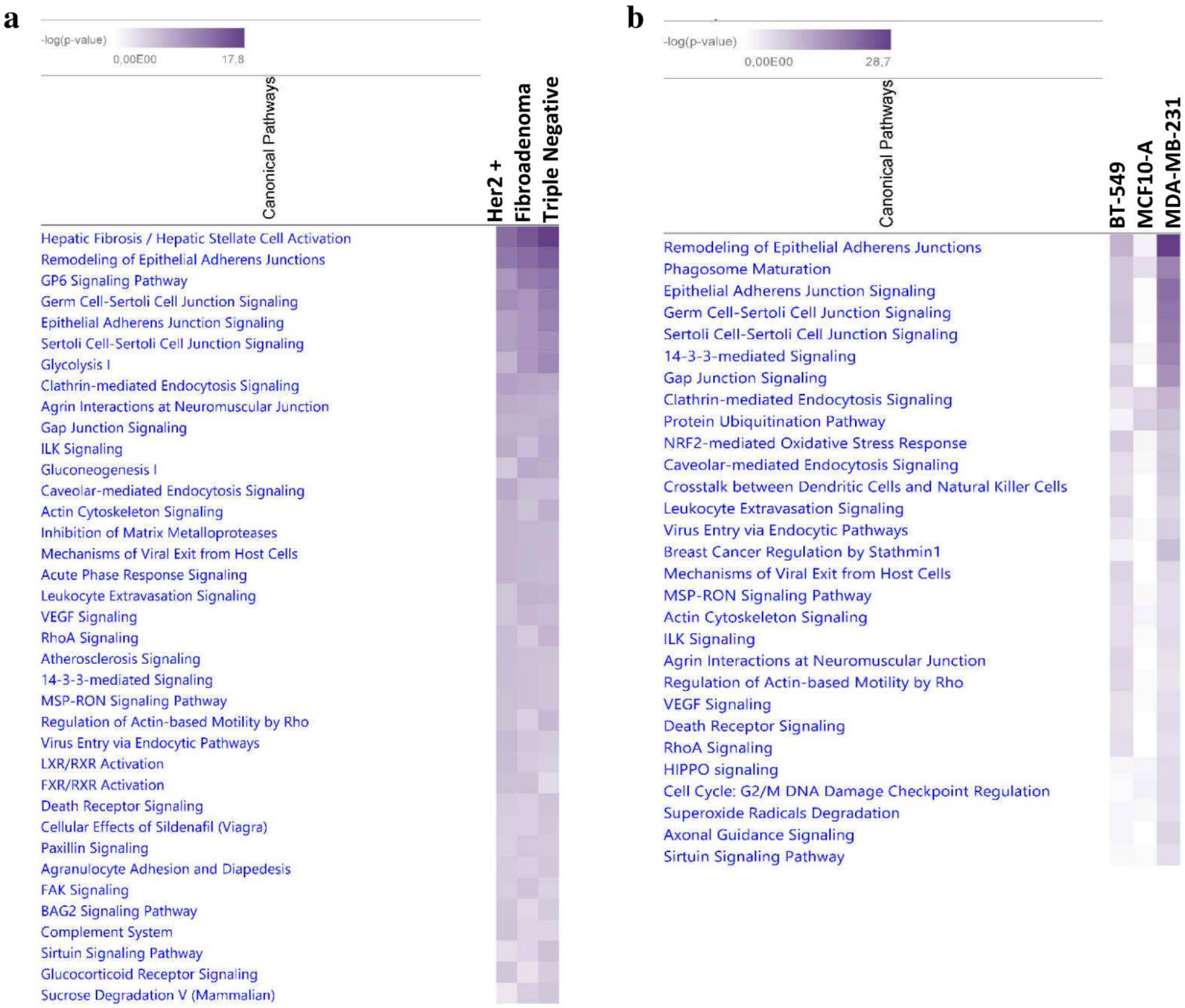

Figure S2. Pathway analysis of exosome cargoes. a) and b) Heatmap of the EVs from patient–derived and model breast cells enriched pathways, respectively. Enrichment score threshold ≥ 5 (Fisher's Exact right-tailed test).

Figure S3 attached to Figure 3

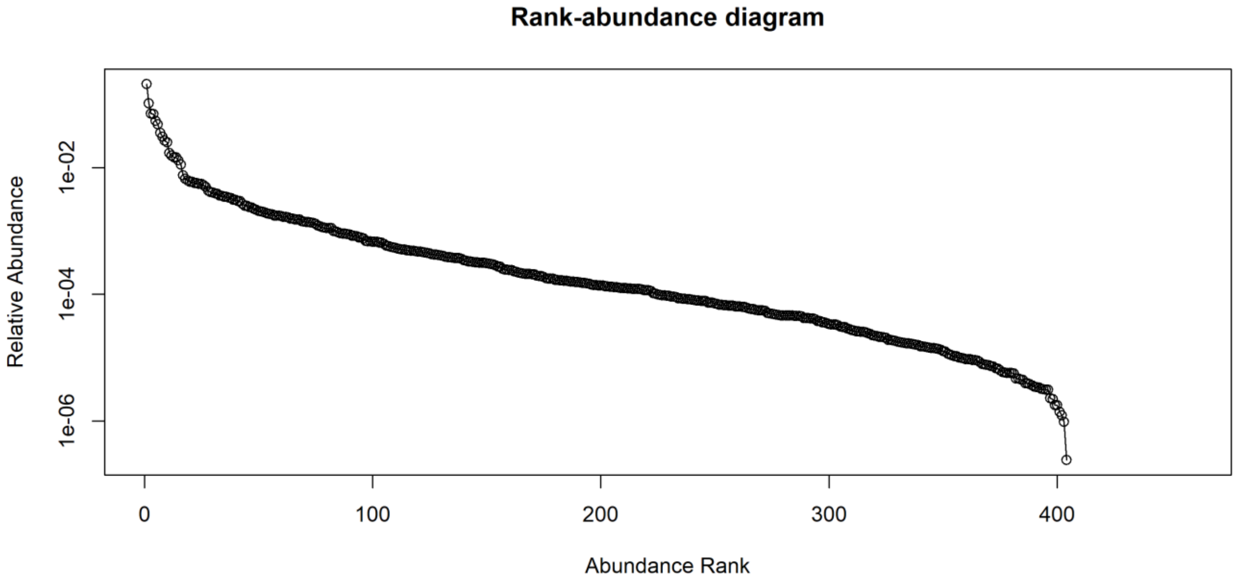

Figure S3. LFQ intensity protein rank-abundance curve. The y-axis is displayed on a log-scale, MaxQuant output.
